# Genome-Wide Association Studies of Embryogenic Callus Induction Rate in Peanut (*Arachis hypogaea* L.)

**DOI:** 10.3390/genes15020160

**Published:** 2024-01-26

**Authors:** Dandan Luo, Lei Shi, Ziqi Sun, Feiyan Qi, Hongfei Liu, Lulu Xue, Xiaona Li, Han Liu, Pengyu Qu, Huanhuan Zhao, Xiaodong Dai, Wenzhao Dong, Zheng Zheng, Bingyan Huang, Liuyang Fu, Xinyou Zhang

**Affiliations:** 1 School of Life Sciences, Zhengzhou University, Zhengzhou 450001, China; 2 Institute of Crop Molecular Breeding, Henan Academy of Agricultural Sciences, Zhengzhou 450002, China; 3 Key Laboratory of Oil Crops in Huang-Huai-Hai Plains, Ministry of Agriculture, Zhengzhou 450002, China; 4 Henan Provincial Key Laboratory for Oil Crops Improvement, Zhengzhou 450002, China; 5 The Shennong Laboratory, Zhengzhou 450002, China; 6 School of Agricultural Sciences, Zhengzhou University, Zhengzhou 450001, China; 7 National Innovation Center for Bio-Breeding Industry, Xinxiang 453500, China

**Keywords:** peanut, CIR, GWAS, candidate gene

## Abstract

The capability of embryogenic callus induction is a prerequisite for in vitro plant regeneration. However, embryogenic callus induction is strongly genotype-dependent, thus hindering the development of in vitro plant genetic engineering technology. In this study, to examine the genetic variation in embryogenic callus induction rate (CIR) in peanut (*Arachis hypogaea* L.) at the seventh, eighth, and ninth subcultures (T7, T8, and T9, respectively), we performed genome-wide association studies (GWAS) for CIR in a population of 353 peanut accessions. The coefficient of variation of CIR among the genotypes was high in the T7, T8, and T9 subcultures (33.06%, 34.18%, and 35.54%, respectively), and the average CIR ranged from 1.58 to 1.66. A total of 53 significant single-nucleotide polymorphisms (SNPs) were detected (based on the threshold value −log_10_(*p*) = 4.5). Among these SNPs, SNPB03-83801701 showed high phenotypic variance and neared a gene that encodes a peroxisomal ABC transporter 1. SNPA05-94095749, representing a nonsynonymous mutation, was located in the *Arahy.MIX90M* locus (encoding an auxin response factor 19 protein) at T8, which was associated with callus formation. These results provide guidance for future elucidation of the regulatory mechanism of embryogenic callus induction in peanut.

## 1. Introduction

Cultivated peanut (*Arachis hypogaea* L.) is among the most economically important oil, food, and feed crops worldwide [1]. The seeds are rich in protein and oleic acid, which can regulate human physiological functions and promote growth and development. The seeds have a high nutritional value and are readily absorbed and utilized [2]. In recent years, transgenic technology [3,4] has been increasingly widely applied in crop genetics and breeding, which has not only reduced the impact of diseases and pests on peanut yield and quality, but also overcome the problem of resistance in traditional breeding. Tissue culture is a basic technology essential for transgenic breeding and verification of gene function. However, in addition to the strong genotype dependence of the in vitro culture of peanut, the plant regeneration rate is low, which greatly limits biotechnology-based breeding. Therefore, there is an urgent need to elucidate the genetic mechanism controlling embryogenic callus induction in peanut to enhance the efficiency of genetic improvement and breeding.

Tissue culture response refers to the process by which any organ, tissue, or cell of a plant develops into a complete plant in a specific environment. In 1958, Steward et al. [5] proposed that plants produced disorganized cell clusters in response to adversity stress, which had the ability to develop into complete plants. Embryogenic callus induction is a crucial step in tissue culture and the initial process of somatic cells regaining totipotency, which is caused by changes in endogenous plant hormone concentrations [6,7]. *ARF7* and *ARF19* regulate the callus formation of *Arabidopsis thaliana* by activating the promoter and expression of *LBD16*, *LBD17*, *LBD18*, and *LBD29* [8] in the absence of exogenous hormones. Inhibiting the expression of these genes inhibits callus formation. Interestingly, root explants of the Arabidopsis *aux1* mutant do not form callus on the standard medium, but callus formation is initiated with an increase in the auxin concentration in the medium. Moreover, the induction and differentiation of embryogenic callus show similar histological characteristics to those of the root meristem [9,10]. *WIND1* can promote callus formation and shoot regeneration by upregulating the expression of *ESR1* [11]. *ESR1* is a differentially expressed gene, which is encoded by an APETALA 2/Ethylene Response Factor (AP2/ERF) transcription factor in Arabidopsis, and high abundance of the WIND1 protein can induce cell dedifferentiation [12,13].

The formation of plant embryogenic callus is controlled by heredity, which can be qualitative [14] or quantitative [15,16,17]. Therefore, the elucidation of the mechanism of callus formation is of considerable importance for the optimization of plant in vitro culture systems. The GWAS approach can help determine the number of loci controlling the genetic variation of complex traits and predict candidate genes [18]. Quantitative trait loci (QTLs) controlling callus differentiation have been identified in many crops, such as rice [19], *Populus euphratica* [20], soybean [21], maize [22], and wheat [23]. In recent decades, with the development of diverse molecular markers, more accurate high-throughput sequencing technology has been applied to peanut genetics and breeding [24,25]. In the present study, we compared the genotype and callus induction rate (CIR) of peanut varieties, constructing a high-density SNP-array genetic map based on the whole-genome resequencing of 353 peanut accessions, with the aim of identifying germplasm resources with a high CIR to expand the efficiency of the genetic transformation of peanut. The results are an important foundation for improvement in the genetic transformation and germplasm conservation of peanut.

## 2. Materials and Methods

### 2.1. Plant Material

A panel of 353 peanut accessions from 26 countries was used in this study, which represented five botanical varieties and two irregular types. The irregular morphologies were formed by the hybridization of different botanical varieties, which were classified as irregular types [26]. These accessions comprise 44 irregular *fastigiata*, 100 irregular *hypogaea*, 26 subsp. *fastigiata* var. *fastigiata*, 2 subsp. *fastigiata* var*. peruviana*, 84 subsp. *fastigiata* var*. vulgaris*, 12 subsp. *hypogaea* var*. hirsuta*, and 85 subsp. *hypogaea* var*. hypogaea* accessions (Table S1). The CIR of each accession was observed and recorded; however, only data for 335 accessions were obtained and analyzed, as some accessions were excluded due to contamination.

### 2.2. Embryogenic Callus Induction

Mature pods of the 353 accessions were artificially shelled, and then clean and mature seeds without plaque were selected. The seeds were disinfected with 75% ethanol for 30 s, followed by 1% (*w/v*) NaClO solution for 8 min, and finally rinsed five times with sterile water. Next, the testa was peeled off with sterile tweezers after the seeds were removed from the water; then, the embryonic axes were excised and inoculated on a SEM medium composed of MS salts, B5 vitamins, 0.088 M sucrose, 12.4 µM picloram, and 0.8% agar (the pH was adjusted to 5.8) [27]. The embryos were cultured on the medium and were subcultured at 4-week intervals. Each subculture of each accession comprised five replicates, and the experiment was repeated three times.

### 2.3. Phenotypic Identification and Statistics

After the sixth subculture, the physiological status of the callus began to stabilize. The number of calli was recorded in each of the seventh, eighth, and ninth subcultures (T7, T8, and T9, respectively). The CIR was calculated as follows [28]:

CIR = *N*/*N*_0_

where *N* is the final callus number and *N*_0_ is the number of inoculations. The CIR value was used for GWAS analysis (Table S1). All phenotypic data were analyzed using Graph Pad Prism 7 and IBM SPSS Statistics 25, including correlation analysis, analysis of variance (ANOVA), and frequency distribution analysis. All phenotypic evaluations were conducted by the same person to maintain consistency between tissue-culture experiments and phenotypic evaluations, and to minimize artificial errors caused by different operational behaviors.

### 2.4. Genome-Wide Association Study

DNA extraction, library preparation, and resequencing were performed as described in detail in a previous study [26]. Whole-genome resequencing of the 353 peanut accessions was conducted using the Illumina HiSeq platform (San Diego, CA, USA). A total of 864,179 single-nucleotide polymorphisms (SNPs) and 71,052 insertions/deletions (InDels) were obtained after quality control. The mixed linear model (MLM) GWAS method [29] was implemented in R package GAPIT (v 3.0) based on the high-density markers. In this study, the significance of traits and markers in association was determined using a threshold of −log_10_(*p*) = 4.5. Manhattan and Q-Q plots were generated using the ‘qqman’ R package [30]. We used the 200 kb genomic regions upstream and downstream of the SNPs that were significantly associated with the peanut CIR as the candidate genomic region, and we used heterotetraploid cultivated peanut genomes for functional annotation. Based on previous reports, we predicted candidate genes associated with embryogenic callus induction in peanut.

## 3. Results

### 3.1. Callus Formation

**Figure 1:**
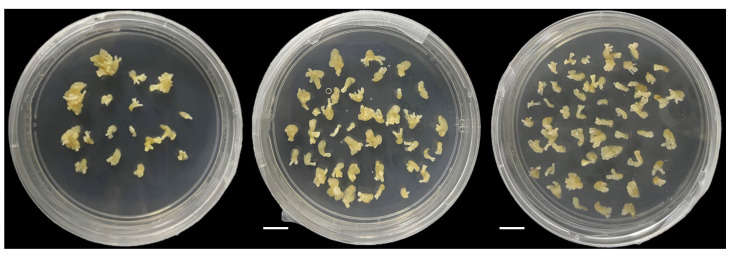
Growth status of peanut callus at the seventh (T7, **left**), eighth (T8, **center**), and ninth (T9, **right**) subcultures.

**Figure 2:**
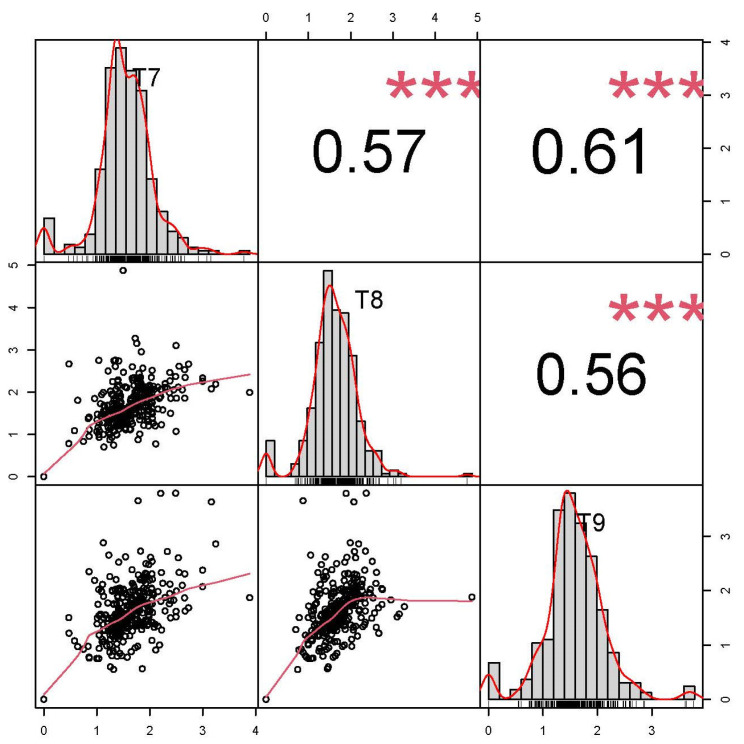
Correlation between callus induction rate in peanut at the T7, T8, and T9 subcultures. The correlation coefficients are presented above the diagonal, and the distributions of the number of calli data at T7, T8, and T9 are presented on and below the diagonal. *** *p* < 0.001.

On the germination medium, 324 of the 335 peanut accessions formed callus, and the growth status of the callus was recorded at T7, T8, and T9 (Figure 1). The CIR phenotype values of the 335 accessions were normally distributed at T7, T8, and T9, with skewness ranging from −0.17 to 0.36 and kurtosis ranging from 2.76 to 4.15 (Figure 2, Table S2). The average CIR ranged from 1.58 to 1.66, and the coefficient of variation was 33.06%, 34.18%, and 35.54% at T7, T8, and T9, respectively (Table S2). A significance analysis showed that the CIR was significant, and a moderately high correlation coefficient was observed between embryogenic callus induction in the three subcultures (0.56 to 0.61) (Figure 2).

**Figure 3:**
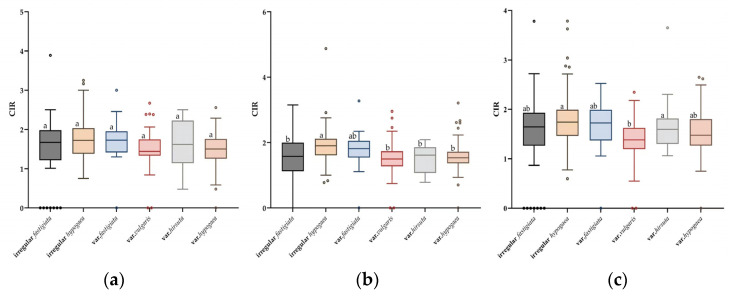
Analysis of variance of differences in callus induction rate (CIR) among six peanut botanical varieties and irregular types at the T7, T8, and T9 subcultures. Boxplots show the variation in the CIR in each peanut type at T7 (**a**), T8 (**b**), and T9 (**c**). Different lowercase letters above the boxes indicate a significant difference within a subculture (*p* ≤ 0.05; Tukey’s HSD test).

Given that only two accessions of subsp. *fastigiata* var. *peruviana* were included in the study panel, the phenotypic variation of the remaining four varieties and two irregular types was analyzed. At T7, the CIR varied among the six types, but the differences were not significant. The CIR values of the six types were ranked in the following order: var. *hirsuta* > irregular *hypogaea* > irregular *fastigiata* > var. *fastigiata* > var*. vulgaris* > var*. hypogaea* (Figure 3a). However, at T8 and T9, significant differences in CIR were observed among the six types, among which the highest CIR was recorded for the irregular *hypogaea* type, and the lowest CIR was that of var*. vulgaris* (Figure 3b,c).

### 3.2. Genome-Wide Association Study and SNP Detection

**Figure 4:**
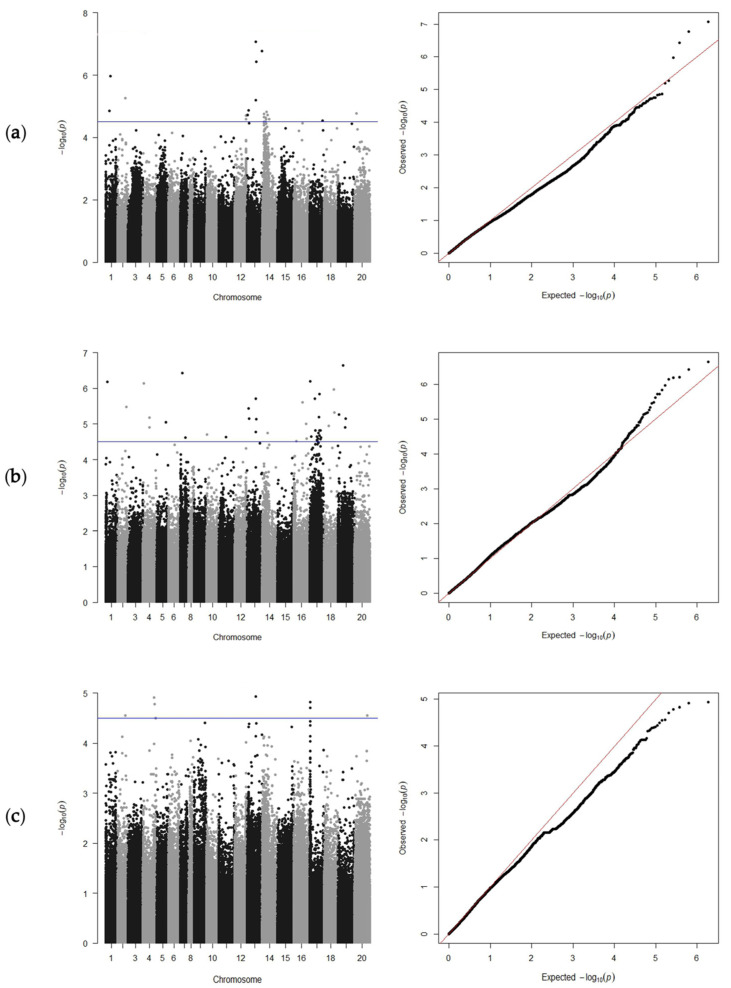
Manhattan diagrams (left) and Q-Q plots (right) of the peanut callus induction rate at the T7, T8, and T9 subcultures. Plots are presented for T7 (**a**), T8 (**b**), and T9 (**c**). The blue line indicates the genome-wide significance threshold (−log_10_(*p*) = 4.5).

Using the CIR of 335 peanut accessions at the three subcultures for GWAS analysis, 53 significant SNPs were detected (Table 1). The GWAS results showed that 23 SNPs were detected at T7, distributed on chromosome (chr) 1, chr2, chr12, chr13, chr 14, chr17, and chr20 (Figure 4a). At T8, 30 SNPs were detected on chr1, chr2, chr4, chr5, chr7, chr10, chr11, chr13, chr14, chr16, and chr17 (Figure 4b). Eight SNPs were detected at T9, which were located on chr2, chr4, chr13, chr17, and chr20 (Figure 4c).

Interestingly, during the three subcultures, SNPs on chr13 formed one small cluster (Figure 4), indicating that it may represent a major QTL, of which only the locus SNPB03-83801701 was detected simultaneously in the three subcultures with a high phenotypic variance (Table 1). Four SNPs overlapped on chr13 at T7 and T8, namely, SNPB03-83801701, SNPB03-91882550, SNPB03-87089142, and SNPB03-17372387 (Table 1). In addition, two SNPs were localized within genes (Table 2): SNPA05-94095749 was localized on chr5 at position 94095749, which resulted in a nonsynonymous mutation in the gene *Arahy.MIX90M*; and SNPB07-5025554 was located on chr17 at position 5025554 in the gene *Arahy.226PX6*, which encodes the uncharacterized protein LOC100778027 isoform X2.

### 3.3. Analysis of Candidate Genes for SNP Loci Associated with CIR in Peanut

Genomic regions within 200 kb upstream and downstream of the significant SNPs were selected as candidate regions. Based on the SNPs significantly associated with CIR and functional annotations in these regions from the peanut reference genome, 600 annotated genes were obtained (Table S3). Thirty-six genes were potentially associated with embryogenic callus induction (Table 2). At T7, 17 genes were located near the nine significant SNPs, of which the most highly significant SNP was on chr13 at position 83801701, corresponding with the gene *Arahy.LC8K5G* that encodes a peroxisomal ABC transporter 1. At T8, five significant SNPs associated with CIR were detected, of which three SNPs were located on chr13. These SNP regions contained genes that affect embryogenic callus induction, and which are directly involved in callus formation or related developmental processes, and they comprised a peroxisomal ABC transporter 1, Pentatricopeptide repeat (PPR) superfamily protein, SAUR-like auxin-responsive protein, MYB transcription factor, and Homeodomain-like transcriptional regulator. The candidate region centered on the SNPA05-94095749 locus contained six candidate genes on chr05, of which four encoded an auxin response factor. At T9, the locus SNPB03-87089142 had the highest phenotypic variance (Table 1).

## 4. Discussion

This study is the first to use GWAS to explore the genetic basis of embryogenic callus induction in peanut. Here, we presented CIR data from 335 peanut accessions in three subcultures, identified the candidate genomic regions, and predicted genes associated with callus formation. The results contribute to an improved understanding of the mechanism of embryogenic callus induction and provide insights useful for further studies of tissue culture in peanut.

### 4.1. Induction of Callus from 353 Peanut Genotypes

Many studies have successfully regenerated plants through somatic and organogenic pathways, accelerating research progress in tissue culture and providing a convenient method for the preservation of rare or precious wild-type germplasm and the rapid propagation of superior germplasm in vitro. The source and genotype of explants are the main factors that regulate somatic embryo formation [31,32]. Callus culture is an essential requirement for crop genetic transformation, but most studies of peanut callus culture to date have focused on comparisons among a small number of varieties, thus knowledge of the genetic mechanism of peanut callus induction is incomplete. The CIR is the most intuitive trait that characterizes the strength of callus induction ability [33]. To explore the genetic basis of variation in callus differentiation, we studied the CIR of 353 peanut accessions representing five botanical varieties and two irregular types, and identified candidate genomic regions and associated genes that control callus differentiation. The present study used the largest number of peanut genotypes of any published study to date to evaluate single-callus formation.

The present data showed that the CIR in the three subcultures was continuous and normally distributed (Figure 2), indicating that CIR was controlled by multiple loci in the study population. In addition, differences in CIR were observed among different botanical varieties and irregular types, for which the difference was not significant at T7, but was significant at T8 and T9 (Figure 3). This variation may be caused by the unstable physiological state of the calli in the early period of embryogenic callus induction in peanut. In addition, the CIR phenotype values in the three subcultures were strongly correlated (Figure 2). These results reflected that the CIR phenotype may be partially controlled by the same genetic factors. In a previous report, the CIR of three peanut market types was ranked as Spanish type > Valencia type > Virginia type [34]. In the present study, at T8 and T9, the CIR of the irregular *hypogaea* type was the highest, and that of var. *vulgaris* was the lowest, which is inconsistent with previous findings. This difference may reflect the different numbers and genotypic representations of materials in the study. Nevertheless, the results confirm that the population structure plays an important role in regulating callus formation.

### 4.2. GWAS Analysis

To date, many studies have been conducted on genes associated with embryogenic callus induction in plants [35,36,37]. In the present study, several significant SNPs merit discussion in detail (Table 2). The peak SNPB03-83801701 was detected in each of the three subcultures and was located 53 kb from *Arahy.LC8K5G*, which encodes a peroxisomal ABC transporter 1. Peroxisome transporters are crucial for the synthesis of certain bioactive molecules, such as docosahexaenoic acid in mammals and jasmonic acid [38]. This protein may regulate plant growth and development by mediating the synthesis of specific bioactive molecules [39]. 

In the present study, a gene with nonsynonymous mutations was detected in proximity to SNPA05-94095749, namely, *Arahy.MIX90M*, which encodes ARF19 [40], a transcriptional activator of auxin early-response genes. In Arabidopsis, *ARF19* regulates lateral root formation by activating *LBD/ASL* genes, reflecting that auxin-induced callus formation has similar characteristics to the root formation metabolic pathway [41]. Moreover, it was observed that the candidate genes *Arahy.SI4FKG*, *Arahy.HDV1A7*, *Arahy.F6IJX6*, *Arahy.X8R2JI*, and *Arahy.K8JH1A* encode ARF19, an auxin canalization protein, and ARF19-like isoform X1. Auxin signaling activates the expression of downstream genes and regulates plant tissue meristem and embryonic development by mediating these auxin transcription factors and auxin channel protein genes [42]. Furthermore, we located several genes on chr13 that were significantly associated with embryogenic callus induction, most of which were associated with a PPR superfamily protein, Homeodomain-like transcriptional regulator, and Saur-like auxin responsive protein. 

The peak SNPB03-17372387 was located 119 kb from *Arahy.2NE4Q7*, which encodes a SAUR-like auxin-responsive protein family member. *SAUR* genes are the largest early auxin-responsive gene family in plants [43]. Active auxin can rapidly induce the expression of *SAUR* genes [44]. A *SAUR* gene was first identified in soybean hypocotyls [45], which had been confirmed to play a role in different processes in plant growth, development, and stress response [46,47,48]. Therefore, it was speculated that candidate genes may affect plant tissue differentiation by regulating cell growth and development. The SNP marker SNPA05-94095749 is located in *Arahy.KFS3KW*, which encodes a B3 domain-containing VRN1-like transcription factor. VRN1, a critical protein that responds to long-term cold treatment to promote flowering, was detected in the vernalization response of Arabidopsis and is crucial for development in Arabidopsis [49]. Overexpression of VRN1 leads to early flowering and phenotypic abnormalities [50].

Recently, marker–trait associations have been analyzed for embryogenic callus induction in a number of plants; for example, SNPB03-17372387 and SNPB07-124709181 are derived from genes that encode an MYB transcription factor. Ge et al. [51] located *zmMYB138*, which is a nucleus-localized member of the MYB transcription factor family of maize, and determined that *zmMYB138* could promote the formation of maize embryogenic callus through gibberellin signal transduction. The *MYB75* transcription factor in Arabidopsis regulates anthocyanin biosynthesis in *Nicotiana* callus [52]. Significantly, we found that one SNPB07-124709181 was strongly associated with CIR, and this was consistently across genotypes with low or NO callus induction (Figure S1). The linkage analysis suggested that the accessions with CAT/CAT have a high CIR, while C/C have a low or zero CIR at this locus at T7, T8, and T9. Due to the small difference in CIR between the two genotypes of the material, this SNP was not identified at T8 and T9, while there was a significant difference in CIR between the two genotypes at the level of 0.05.

The present study was focused on the prediction of candidate genes associated with embryogenic callus induction. Further functional analysis and verification are needed. At present, although many QTLs of embryogenic callus induction traits have been identified in other crops, there are currently few reports on genes associated with peanut embryogenic callus induction, and the genetic mechanism of peanut embryogenic callus induction remains unclear. The SNPs and candidate genes identified in the current study lay the foundation for further cloning of the functional genes that regulate peanut embryogenic callus induction and analysis of the genetic mechanism of peanut embryogenic callus induction.

## 5. Conclusions

In this study, we conducted the embryogenic callus induction of 353 peanut accessions and observed and scored their phenotypes. The results showed that callus induction might be partially controlled by the same genetic factors. In addition, we identified the genomic regions associated with callus formation and located candidate genes associated with callus formation based on functional annotations.

## Figures and Tables

**Table 1:** Single-nucleotide polymorphisms (SNPs) significantly associated with peanut callus induction rate identified at the T7, T8, and T9 subcultures through genome-wide association study.

Subculture	Chr	SNP Location (bp)	Major Allele	Minor Allele	*p*
T7	Arahy.01	44,535,215	T	TA	1.07 × 10^−6^
	Arahy.01	37,905,924	T	TA	1.41 × 10^−5^
	Arahy.02	78,055,673	C	A	5.39 × 10^−6^
	Arahy.12	110,745,152	C	T	2.00 × 10^−5^
	Arahy.12	110,744,821	C	A	2.55 × 10^−5^
	Arahy.13	83,801,701	G	A	8.54 × 10^−8^
	Arahy.13	143,122,895	TA	A	1.70 × 10^−7^
	Arahy.13	91,882,550	G	A	3.69 × 10^−7^
	Arahy.13	87,089,142	G	T	6.30 × 10^−6^
	Arahy.13	17,372,387	G	A	1.36 × 10^−5^
	Arahy.13	7,863,511	CA	C	1.89 × 10^−5^
	Arahy.14	39,653,468	AT	A	1.49 × 10^−5^
	Arahy.14	18,525,892	G	A	1.79 × 10^−5^
	Arahy.14	53,962,810	A	G	1.92 × 10^−5^
	Arahy.14	16,587,777	C	T	2.23 × 10^−5^
	Arahy.14	29,924,017	C	T	2.27 × 10^−5^
	Arahy.14	37,587,465	A	T	2.58 × 10^−5^
	Arahy.14	65,049,781	A	G	2.62 × 10^−5^
	Arahy.14	33,807,861	G	A	3.08 × 10^−5^
	Arahy.14	19,876,608	G	A	3.12 × 10^−5^
	Arahy.14	31,198,728	C	T	3.15 × 10^−5^
	Arahy.17	124,709,181	C	CTA	2.84 × 10^−5^
	Arahy.20	19,417,946	C	CAT	1.73 × 10^−5^
T8	Arahy.01	15,782,356	CA	A	6.59 × 10^−7^
	Arahy.02	85,103,096	CA	C	3.26 × 10^−6^
	Arahy.04	8,623,742	GA	A	7.14 × 10^−7^
	Arahy.04	61,118,310	C	CCAATACTCGTAAAGAGTCTCAGATTTGCCTTGAA	6.65 × 10^−6^
	Arahy.04	64,427,766	AC	A	1.23 × 10^−5^
	Arahy.05	94,095,749	CGGT	C	8.89 × 10^−6^
	Arahy.07	22,858,555	C	CA	3.74 × 10^−7^
	Arahy.07	49,041,776	C	T	2.38 × 10^−5^
	Arahy.10	4,186,469	CA	C	2.00 × 10^−5^
	Arahy.11	67,723,994	C	CT	2.34 × 10^−5^
	Arahy.13	83,801,701	G	A	1.94 × 10^−6^
	Arahy.13	17,372,387	G	A	3.60 × 10^−6^
	Arahy.13	23,273,713	C	A	7.12 × 10^−6^
	Arahy.13	91,882,550	G	A	7.41 × 10^−6^
	Arahy.13	87,089,142	G	T	1.70 × 10^−5^
	Arahy.14	51,951,294	A	G	1.76 × 10^−5^
	Arahy.16	84,573,006	TC	T	2.42 × 10^−6^
	Arahy.16	119,791,828	TA	T	9.75 × 10^−6^
	Arahy.16	122,692,509	GTGC	G	2.60 × 10^−5^
	Arahy.16	24,861,124	C	T	3.02 × 10^−5^
	Arahy.17	5,025,554	GT	G	6.36 × 10^−7^
	Arahy.17	14,468,911	C	T	2.22 × 10^−5^
T9	Arahy.02	78,055,673	C	A	2.82 × 10^−5^
	Arahy.04	105,916,420	C	G	1.22 × 10^−5^
	Arahy.04	111,900,912	C	T	1.67 × 10^−5^
	Arahy.04	119,003,542	G	A	3.20 × 10^−5^
	Arahy.13	83,801,701	G	A	1.18 × 10^−5^
	Arahy.17	6,136,617	C	T	1.51 × 10^−5^
	Arahy.17	6,170,139	C	T	1.96 × 10^−5^
	Arahy.20	118,558,032	C	A	2.78 × 10^−5^

**Table 2:** Single-nucleotide polymorphisms (SNPs) and candidate genes significantly associated with callus induction rate in peanut at the T7, T8, and T9 subcultures.

Subculture	SNP Location (bp)	Chr	Candidate Genes	Distance to SNP (kb)	Functional Annotation
T7	44,535,215	Arahy.01	Arahy.KCTF4J	9 (44,544,077–44,544,971)	SAUR-like auxin-responsive protein family
	37,905,924	Arahy.01	Arahy.6W2W8X	124 (37,777,131–37,781,543)	MYB transcription factor MYB93
	110,744,821	Arahy.12	Arahy.M89JWR	37 (110,782,249–110,794,939)	Pentatricopeptide repeat (PPR) superfamily protein
		Arahy.12	Arahy.46GJXK	67 (110,812,150–110,816,210)	Pentatricopeptide repeat (PPR) superfamily protein
	83,801,701	Arahy.13	Arahy.LC8K5G	53 (83,747,374–83,747,929)	peroxisomal ABC transporter 1
	91,882,550	Arahy.13	Arahy.CH5A50	72 (91,954,110–91,961,683)	Homeodomain-like transcriptional regulator
	17,372,387	Arahy.13	Arahy.I1CX7V	124 (17,245,402–17,248,068)	Pentatricopeptide repeat (PPR) superfamily protein
		Arahy.13	Arahy.2NE4Q7	119 (17,251,821–17,253,819)	SAUR-like auxin-responsive protein family
		Arahy.13	Arahy.4B15T0	126 (17,498,819–17,502,146)	myb transcription factor
	39,653,468	Arahy.14	Arahy.SI4FKG	149 (39,802,122–39,804,507)	auxin response factor 19
	18,525,892	Arahy.14	Arahy.HDV1A7	116 (18,407,047–18,409,728)	auxin canalisation protein
		Arahy.14	Arahy.6YY5F1	93 (18,618,421–18,623,655)	ankyrin repeat-containing protein At5g02620-like isoform X6
	124,709,181	Arahy.17	Arahy.26LA0S	113 (124,822,009–124,824,185)	myb transcription factor
		Arahy.17	Arahy.N9VQ32	156 (124,865,117–124,870,706)	Pentatricopeptide repeat (PPR-like) superfamily protein
		Arahy.17	Arahy.Z51W7G	170 (124,879,592–124,882,521)	Pentatricopeptide repeat (PPR-like) superfamily protein
		Arahy.17	Arahy.A8R8TX	181 (124,890,375–124,892,445)	Pentatricopeptide repeat (PPR-like) superfamily protein
		Arahy.17	Arahy.H6TD7S	183 (124,892,449–124,894,438)	Pentatricopeptide repeat (PPR-like) superfamily protein
T8	94,095,749	Arahy.05	Arahy.F6IJX6	11 (94,082,178–94,084,422)	auxin response factor 19
		Arahy.05	Arahy.X8R2JI	10 (94,084,481–94,084,965)	auxin response factor 19-like isoform X1
		Arahy.05	Arahy.K8JH1A	9 (94,084,986–94,086,080)	auxin response factor 19-like isoform X1
		Arahy.05	Arahy.58VMN1	4 (94,087,242–9,409,170)	acetyl-CoA carboxylase biotin carboxylase subunit
		Arahy.05	Arahy.MIX90M	0 (94,093,951–94,101,641)	auxin response factor 19
		Arahy.05	Arahy.KFS3KW	185 (94,280,364–94,282,288)	B3 domain-containing transcription factor VRN1-like
	83,801,701	Arahy.13	Arahy.LC8K5G	53 (83,747,374–83,747,929)	peroxisomal ABC transporter 1
	17,372,387	Arahy.13	Arahy.I1CX7V	124 (17,245,402–17,248,068)	Pentatricopeptide repeat (PPR) superfamily protein
		Arahy.13	Arahy.2NE4Q7	119 (17,251,821–17,253,819)	SAUR-like auxin-responsive protein family
		Arahy.13	Arahy.4B15T0	126 (17,498,819–17,502,146)	myb transcription factor
	91,882,550	Arahy.13	Arahy.CH5A50	72 (91,954,110–91,961,683)	Homeodomain-like transcriptional regulator
	5,025,554	Arahy.17	Arahy.G50Y2D	102 (4,918,422–4,923,604)	uncharacterized protein LOC100777386 isoform X2
		Arahy.17	Arahy.LA4AI5	82 (4,942,078–4,943,769)	probable WRKY transcription factor 28-like
		Arahy.17	Arahy.226PX6	0 (5,024,406–5,026,216)	uncharacterized protein LOC100778027 isoform X2
T9	83,801,701	Arahy.13	Arahy.LC8K5G	53 (83,747,374–83,747,929)	peroxisomal ABC transporter 1
	6,136,617	Arahy.17	Arahy.6TK8TS	153 (5,981,429–5,983,822)	Pentatricopeptide repeat (PPR) superfamily protein
		Arahy.17	Arahy.I0AKGU	71 (6,066,092–6,068,587)	Pentatricopeptide repeat (PPR) superfamily protein
		Arahy.17	Arahy.6847YK	28 (6,164,347–6,165,237)	Pentatricopeptide repeat (PPR) superfamily protein
	118,558,032	Arahy.20	Arahy.35R4A4	69 (118,488,460–118,489,125)	Myb/SANT-like DNA-binding domain protein

## Data Availability

Data are contained within the article and Supplementary Materials.

## Supplementary Materials

The following supporting information can be downloaded at: https://www.mdpi.com/article/10.3390/genes15020160/s1, Figure S1: Linkage analysis of the peanut callus induction rate (CIR) with SNP at the T7 (**a**), T8 (**b**), and T9 (**c**) subcultures. * *p* < 0.05. **** *p* < 0.0001; Table S1: Features and callus induction rate of 353 *Arachis hypogaea* accessions subjected to whole-genome resequencing; Table S2: Phenotypic variation of callus induction rate for 353 peanut accessions at the T7, T8, and T9 subcultures; Table S3: 600 annotated genes associated with SNPs at the T7, T8, and T9 subcultures.
