# Supplementary material for: Genome-Wide Association Studies of Embryogenic Callus Induction Rate in Peanut (Arachis hypogaea L.)
Source: Genes (Basel). 2024 Jan 26;15(2):160. doi: 10.3390/genes15020160 (PMC10887910; doi:10.3390/genes15020160)
Supplement: Supplementary file 1 [file genes-15-00160-s001.zip › Figure S1.pdf]

SNPB07-124709181

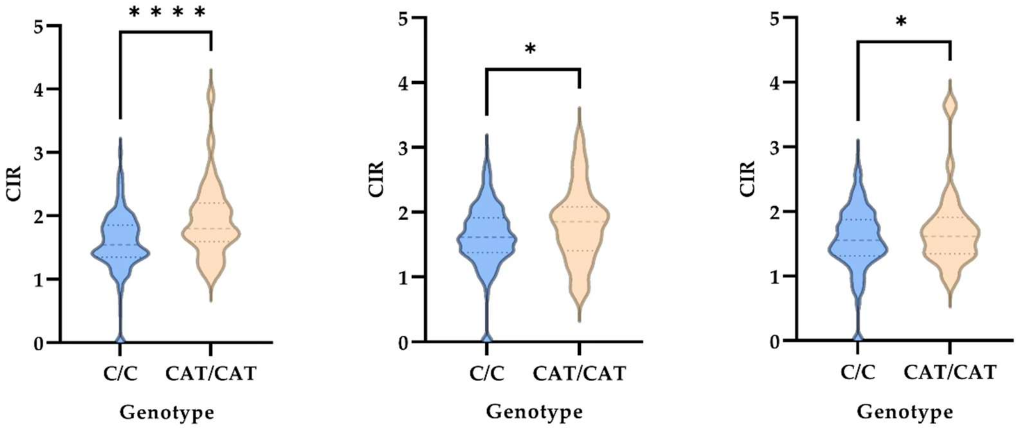

(a) (b) (c)

Figure S1. Linkage analysis of the peanut callus induction rate (CIR) with SNP at the T7 (**a**), T8 (**b**), and T9 (**c**) subcultures. * $p < 0.05$. **** $p < 0.0001$.
